# Deep Learning-Guided Engineering of *Bst* DNA Polymerase Improves LAMP-Based Detection of Foodborne Pathogens

**DOI:** 10.3390/microorganisms14050954

**Published:** 2026-04-23

**Authors:** Haoting Chen, Jingfeng Zhang, Xiaoli Xu, Huang Zhang, Yanlei Chang, Lei Shi, Lichao Zhao

**Affiliations:** 1 College of Food Science, Instrumental Analysis & Research Center, South China Agricultural University, Guangzhou 510642, China; jingfeng.research@gmail.com (J.Z.); xuxiaoli@scau.edu.cn (X.X.); 2 College of Life Sciences, South China Agricultural University, Guangzhou 510642, China; chenhaoting@dhelix.cn; 3 Guangzhou Double Helix Gene Technology Co., Ltd., Guangzhou 510700, China; zhanghuang@dhelix.cn (H.Z.); changyanlei@dhelix.cn (Y.C.); 4 College of Life Sciences, Hebei Agricultural University, Baoding 071000, China

**Keywords:** *Bst* DNA polymerase, foodborne pathogen detection, deep learning, LAMP, fusion protein engineering

## Abstract

Loop-mediated isothermal amplification (LAMP) is a widely used nucleic acid detection method, but its application is often limited by the suboptimal performance of wild-type *Bacillus stearothermophilus* (*Bst*) DNA polymerase. This study employed a combined deep learning and semi-rational design strategy to engineer *Bst* DNA polymerase. High-throughput screening identified the A0A150MFP3 sequence and the L105M mutation, which increased enzymatic activity by 32.92%. Fusion with the CL7 protein generated a CL7-*Bst* mutant with enhanced thermal stability and tolerance to common inhibitors, including 7% (*v*/*v*) ethanol, 0.18‰ (*w*/*v*) SDS, 80 mmol/L NaCl, and 0.8 mmol/L EDTA. Systematic optimization of the LAMP reaction system determined the optimal pH (9.0), enzyme concentration (0.20 U/μL), and temperature (64 °C). When applied to *Escherichia coli* O157:H7 detection, the CL7-*Bst* mutant achieved Tt values of 15.13 and 12.78 for crude and purified DNA, respectively, with a limit of detection of 1 × 10^3^ CFU/mL. In summary, integrating deep learning with semi-rational design and fusion protein engineering yielded a high-performance DNA polymerase that facilitates rapid, sensitive, and field-deployable LAMP-based pathogen detection.

## 1. Introduction

Loop-mediated isothermal amplification (LAMP) is an isothermal nucleic acid amplification technology that operates without the need for complex instrumentation and provides high analytical sensitivity. Since its introduction, it has been widely adopted for the detection of bacterial, viral, and parasitic pathogens [1,2]. *Bacillus stearothermophilus* DNA polymerase (*Bst* DNA polymerase) is the essential enzyme for LAMP reactions. However, the wild-type enzyme possesses suboptimal thermal stability, limited tolerance to inhibitors, and inadequate sensitivity to low-copy targets, which collectively constrain its broader application [3].

Efforts to improve the performance of wild-type *Bst* DNA polymerase have been reported, and multiple patents have been filed concerning its purification and modification. Representative strategies include polyethylene glycol (PEG) conjugation, mutagenesis guided by fluorescence-activated droplet sorting (FADS) technology, and fusion with heterologous proteins [3,4,5,6]. Despite these advances, the demand for more effective modification approaches and enzymes with superior functional properties remains unmet. Therefore, novel modification strategies should be systematically explored to enhance the enzyme’s performance, robustness, and applicability in LAMP-based diagnostics. Traditional research on DNA polymerase molecular engineering has primarily relied on three classical strategies: rational design, irrational design, and semi-rational design. Rational design refers to the use of knowledge regarding the structure and functional properties of the target enzyme, combined with computational modeling and simulation frameworks, to predict mutations, insertions, or deletions aimed at improving enzyme performance. This precise modification approach is highly dependent on a comprehensive understanding of protein structure–function relationships and entails a high technical threshold [7]. Irrational design, by contrast, does not require explicit structural knowledge of the protein. Instead, it simulates natural evolutionary processes to obtain target mutants. However, its major limitation lies in the need to construct a large mutant library [8]. Semi-rational design provides scientists with a smaller and more intelligent mutant library, making it a cost-effective and time-saving strategy superior to irrational design [9]. Its limitation is the requirement for accurate structural information, and there remains room for improvement in terms of efficiency and cost [10]. The shortcomings of these strategies essentially stem from their reliance on traditional computational models or random experimental screening, without fully leveraging the predictive power of deep learning. Therefore, the integration of deep learning technology is expected to overcome the bottlenecks of traditional molecular engineering, enabling the identification of a broader range of enzyme variants and improving both the efficiency and success rate of enzyme modification [8].

The CL7 protein is a mutant form of the CE7 nuclease (Colicin E7 DNase) that has lost nuclease activity while retaining DNA-binding capability [11]. CL7 can be utilized as a fusion tag that can significantly enhance the expression level, solubility, and activity of target proteins. Additionally, the CL7 domain can function as a chaperone, facilitating the correct folding of target proteins [12]. Fusing CL7 to *Taq* DNA polymerase improved thermostability, amplification rate, and template sensitivity [13]. A novel recombinase, FEN1-*Bst* DNA polymerase, was developed by Ye et al. [14], conferring DNA synthesis, strand displacement, and cleavage activity. In previous work, Paik et al. [15] engineered Br512 by fusing the villin HP47 domain to the *N*-terminus of *Bst* DNA polymerase, which demonstrated retained thermostability at 72 °C.

Previous studies have improved LAMP performance primarily by optimizing primer design. However, the reaction system conditions (component concentrations and temperature) are also critical: even minor deviations can cause nonspecific amplification or reduced enzyme activity [4]. Furthermore, different enzymes or targets may require distinct reaction conditions. Notably, little research has focused on optimizing LAMP conditions for crudely extracted samples, which can limit the effectiveness of on-site pathogen detection.

In response to these issues, we combined deep learning with semi-rational design to engineer *Bst* DNA polymerase. A deep learning model was used for high-throughput screening of candidate sequences, identifying A0A150MFP3 as a promising variant for further engineering. We then applied site-directed mutagenesis and fusion protein techniques to create a CL7-*Bst* mutant with enhanced enzymatic activity, thermal stability, and inhibitor tolerance relative to the wild-type. Finally, we systematically optimized the LAMP reaction parameters for the mutant enzyme and evaluated its application in foodborne pathogen detection, using *Escherichia coli* O157:H7 as a representative target, including detection from crudely extracted samples.

## 2. Materials and Methods

### 2.1. Bacterial Strains

*Geobacillus stearothermophilus* was obtained from the Guangdong Microbial Culture Collection Center (GDMCC). The plasmid pET-28a was supplied by Prof. Junfang Lin at the College of Food Science, South China Agricultural University. *Escherichia coli* DH5α and *Escherichia coli* BL21(DE3) were purchased from Sangon Biotech (Shanghai, China). *E. coli* O157:H7 ATCC 35150 and *Salmonella enterica* serovar *Typhimurium* (*S. Typhimurium*) ATCC 14028 were preserved by the research group.

### 2.2. Protein Selection

Protein sequences meeting the filtering criteria, including enzyme name, gene type, and protein sequence length, were retrieved from the UniProt database using Python v.3.7.6, and the relevant files were generated. Substrate structural information was obtained from the PubChem compound database using the English name of the substrate, and the corresponding simplified molecular input line entry specification (SMILES) strings were recorded. The retrieved enzyme sequences and substrate information were then subjected to high-throughput *k_cat_* prediction using the DLKcat deep learning tool, which combines a graph neural network (GNN) for substrate representation and a convolutional neural network (CNN) for protein representation. In this model, substrate structures were represented as molecular graphs converted from SMILES strings, while protein sequences were split into overlapping continuous amino acid subsequences composed of *n* elements and converted into vectors. The attention mechanism was used to extract and visualize important signals from the neural networks, and model parameters, including the number of vertices in the r-radius substrate subgraph, the feature vector dimension of n-gram amino acids, the number of time steps in the GNN, and the number of layers in the CNN, were optimized using the training dataset. Finally, automated Python scripts were used to batch simulate all protein sequences obtained in step (1) with the substrate and predict the corresponding *k_cat_* values, following the published DLKcat workflow [16].

### 2.3. DNA Extraction

Total DNA was extracted as follows: 3 mL of an overnight bacterial culture was centrifuged at 10,000× *g* rpm for 1 min, and the supernatant was discarded. The pellet was resuspended in 180 μL Lysozyme solution by pipetting and incubated at 37 °C in a metal bath for 30 min. After adding 20 μL Proteinase K with thorough pipette mixing, 250 μL Buffer GB was introduced and mixed similarly, followed by 10 min incubation at 70 °C in a metal bath.

Subsequently, 180 μL absolute ethanol was added with vortex mixing, and the mixture was briefly centrifuged to collect residual liquid from tube walls. The entire solution was transferred to a FastPure gDNA Mini Columns III (Vazyme, Nanjing, China) adsorption column and centrifuged at 12,000 rpm for 1 min (flow-through discarded). The column was washed with 500 μL Buffer PB (centrifuged at 12,000 rpm for 1 min, flow-through discarded), followed by two washes with 600 μL Buffer PW under identical centrifugation conditions. The column was recentrifuged at 12,000 rpm for 2 min in an empty collection tube. Finally, DNA was eluted by applying 50 μL pre-warmed (55 °C) ddH_2_O to the center of the membrane, incubating at room temperature for 2 min, and centrifuging at 12,000 rpm for 1 min into a sterile 1.5 mL tube. This elution step was repeated once. DNA concentration was measured using NanoDrop One (1 μL aliquot), and the product was stored at −20 °C.

### 2.4. Vector Construction

DNA fragments (for sequences A0A023CMU9, A9X455, and v5.9; Milligan et al., 2018) [17] were synthesized by Sangon Biotech Co., Ltd. (Shanghai, China) and each was ligated into the pET-28a vector. The A0A150MFP3 DNA fragment was amplified with gene-specific primers (Table S1). The 50 μL PCR mixture was prepared containing 2 μL of DNA, 25 μL of 2× Phanta Flash Master Mix (Dye Plus) (Vazyme, Nanjing, China), 21 μL of ddH_2_O, and 1 μL of each primer. Amplification was performed under the following conditions: initial denaturation at 94 °C for 5 min; 30 cycles of denaturation at 95 °C for 30 s, annealing at 55 °C for 30 s, and extension at 72 °C for 10 s; followed by a final extension at 72 °C for 10 min. The amplified products were purified using the QIAquick PCR Purification Kit (QIAGEN, Hilden, Germany). The products were ligated into the pET-28a vector using *EcoR* I and *Xho* I digestion followed by T4 DNA ligase, and the four vectors were transformed into *E. coli* strain BL21.

### 2.5. Mutant Site Selection

The tertiary structure of A0A150MFP3 DNA polymerase large fragment was predicted using the SWISS-MODEL online server (https://swissmodel.expasy.org/, accessed on 22 August 2025). Model validation was performed through the SAVES server (https://saves.mbi.ucla.edu/, accessed on 22 August 2025) and Discovery Studio 4.0. Active sites were predicted via COACH (https://zhanggroup.org/COACH/, accessed on 22 August 2025) and HotSpot-Wizard (https://loschmidt.chemi.muni.cz/hotspotwizard/, accessed on 22 August 2025). Alanine scanning (in Discovery Studio) was performed on all residues within 3 Å of the ligand; key mutations with ΔΔG > 0.5 kcal/mol were flagged for further analysis. Saturation mutagenesis was then performed on these critical residues to screen beneficial variants. Twenty mutation hotspots were prioritized based on consensus predictions from COACH and HotSpot-Wizard. Primers for the 20 mutants were designed using the QuikChange Primer Design tool (https://www.agilent.com/store/primerDesignProgram.jsp, accessed on 22 August 2025). Detailed primer information is provided in Table S1. The plasmid pET28a-A0A150MFP3 (hereafter referred to as pET28a-*Bst*) vector was then transformed into *E. coli* strain BL21 and verified by DNA sequencing.

### 2.6. CL7-Bst Mutant Vector Construction

Coding sequence of CL7 protein derived from *E. coli* and the sequence of Linker were synthesized by Sangon Biotech Co., Ltd. (Shanghai, China). The CL7-Linker fragment was ligated into the pET-28a vector using *BamH* I and *Sac* II digestion followed by T4 DNA ligase. In the pET28a-*Bst* mutant plasmid, the nucleotide at position 313 of the *Bst* gene was mutated from thymine (T) to adenine (A).

### 2.7. Expression and Purification of Bst DNA Polymerases

*E. coli* strain BL21 carrying the plasmids was cultured in liquid Luria–Bertani (LB) medium with 50 μg/mL kanamycin overnight at 37 °C with shaking. Then 5 mL of culture was added to 1 L of liquid LB medium and incubated to OD_600_ = 0.6–0.8 at 37 °C with shaking. Then, the culture was subjected to shake-flask fermentation with 0.2 mmol/L isopropyl β-D-1-thiogalactopyranoside (IPTG) overnight at 18 °C with shaking. The cells were then collected via centrifugation at 4000× *g* for 20 min and stored at −80 °C. The cell pellets were resuspended in 30 mL lysis buffer (containing 50 mM Tris–HCl pH 8.0, 50 mM NaCl, 5% glycerol), then they were sonicated (3 s ON, 5 s OFF, 165 W) for 20 min in an ice bath. The lysate was then centrifuged at 36,000× *g* for 30 min at 10 °C to obtain a clarified lysate.

Purification of the target protein was performed using nickel affinity chromatography followed by ion exchange chromatography [6]. *Bst* DNA polymerases were loaded onto a 1 mL HiTrap^TM^ Chelating HP column (Cytiva, Hangzhou, China) pre-equilibrated with lysis buffer. Subsequently, the column was washed with 25 mL of lysis buffer. Bound proteins were fractionated through linear gradient elution (50–500 mM imidazole in lysis buffer), with target protein eluting at approximately 100 mM imidazole. Target protein-containing fractions were pooled and loaded onto a 5 mL HiTrap™ Capto™ Q column (Cytiva Hangzhou, China) pre-equilibrated with lysis buffer. A steep NaCl gradient (100 mM to 1 M) in elution buffer (50 mM Tris–HCl, pH 8.0; 5% glycerol) was applied, resulting in recombinant protein elution at ~200 mM NaCl.

Eluates containing the enzyme were collected and dialyzed against dialysis buffer (10 mM Tris-HCl, 50 mM KCl, 0.1 mM EDTA, 50% glycerol, pH 8.0) at 4 °C for 12 h. The dialyzed recombinant enzyme was subsequently solubilized in storage buffer (10 mM Tris-HCl, 50 mM KCl, 0.1 mM EDTA, 2 mM DTT, 0.15% Triton X-100, 50% glycerol, pH 8.0) at a 1:1 (*v*/*v*) ratio and stored at −20 °C. All fractions obtained throughout the purification process were analyzed by sodium dodecyl sulfate-polyacrylamide gel electrophoresis (SDS-PAGE). Protein concentrations of chimeric *Bst* DNA polymerases were quantified using a BCA Protein Assay Kit (Tiangen Biotech, Beijing, China).

### 2.8. Real-Time LAMP Reaction System

Each 25 μL real-time LAMP reaction contained 2.5 μL of 10× buffer (final: 20 mM Tris-HCl pH 8.8, 10 mM KCl, 10 mM (NH_4_)_2_SO_4_, 2 mM MgSO_4_, 0.1% Triton X-100), 0.8 μM each inner primer (FIP, BIP), 0.4 μM each loop primer (LF, LB), 0.2 μM each outer primer (F3, B3), 0.5 μL of additional MgSO_4_, 3.5 μL of 10 mM dNTP mix, 1 μL of *Bst* DNA polymerase, 1.25 μL of 10× SYTO-9 fluorescent dye, and 2 μL of DNA template. Reactions were incubated at 64 °C for 40 min on a StepOnePlus^TM^ Real-Time PCR System (Applied Biosystems, Foster City, CA, USA), with fluorescence signals acquired at 1 min intervals to monitor amplification progress. All experiments were performed in triplicate; representative amplification curves are shown.

### 2.9. Amplification Performance of Bst DNA Polymerases in LAMP Reaction System

Genomic DNA extracted from *S. typhimurium* ATCC 14028 served as the template. The genomic DNA was isolated using a Genomic DNA Extraction Kit (Guangzhou Double Helix Gene Technology Co., Ltd., Guangzhou, China) according to the manufacturer’s protocol. LAMP primers targeting the *invA* gene were designed using PrimerExplorer V5 (Eiken Chemical Co., Tokyo, Japan). Reaction mixtures were subsequently assembled to evaluate enzymatic activity of recombinant proteins in the supernatants of the following expression constructs: pET28a-*Bst*, pET28a-A9X455, pET28a-A0A023CMU9, and pET28a-v5.9.

### 2.10. DNA Polymerase Activity Assay

Polymerase activity was determined according to established methodologies [18] using a CFX96 Touch^TM^ Real-Time PCR System (Bio-Rad, Hercules, CA, USA). The initial fluorescence increase rate was calculated as the slope of fluorescence intensity versus time during the linear amplification phase, which exhibits direct proportionality to enzyme concentration. For samples, the initial fluorescence increase rate of polymerase-catalyzed reactions was measured and converted to enzymatic activity units based on a standard calibration curve.

One unit of DNA polymerase activity is defined as the amount of enzyme required to incorporate 10 nmol of dNTPs into acid-insoluble precipitates within 30 min at 60 °C [19].

### 2.11. Optimization of LAMP Reaction System Composition and Concentration

The reaction system was optimized by varying the concentration of the following components: Tris-HCl (10–80 mmol/L), KCl (0–140 mmol/L), (NH_4_)_2_SO_4_ (0–60 mmol/L), Triton X-100 (0.1–0.6%), MgSO_4_ (4–14 mmol/L), dNTPs (0.4–2.4 mmol/L), and SYBR Green I (0.2×–1×).

### 2.12. Thermal Stability

The recombinant protein was incubated at 20–90 °C (in 10 °C increments) for 2 h, then immediately cooled on ice. The residual enzyme activity was detected according to the method described in Section 2.9. The enzyme activity without incubation was set as 100%, then the relative enzyme activity was calculated.

### 2.13. Determination of the Inhibitor Tolerance

In order to examine the tolerance of CL7-*Bst* mutant to inhibitors, Real-time LAMP assays were performed in the presence of various potential inhibitors at different concentrations: ethanol (0–8% *v*/*v*), SDS (0–0.2‰ *w*/*v*), NaCl (0–140 mmol/L), and EDTA (0–1 mmol/L). The tolerance of the CL7-*Bst* mutant was compared to that of a commercial *Bst* DNA Polymerase Large Fragment (Vazyme, Nanjing, China) under these conditions.

### 2.14. Determination of Optimal Concentration of CL7-Bst Mutant Recombinant Protein

Using the reaction system optimized in Section 2.11, the final concentration of CL7-*Bst* mutant recombinant protein was adjusted to 0.08, 0.12, 0.16, 0.20, 0.24, 0.28, and 0.32 U/μL, respectively. Ultrapure water served as the negative control.

### 2.15. Determination of Optimal Reaction Temperature

Using the previously optimized conditions (Section 2.11 and Section 2.14), LAMP reactions were performed at temperatures from 61 °C to 65 °C (1 °C increments). The amplification efficiency at each temperature was measured to determine the optimal reaction temperature for the CL7-*Bst* LAMP system.

### 2.16. Determination of Optimal Reaction pH

Using the previously optimized conditions (Section 2.11 and Section 2.14), the reaction pH was varied between 7.0 and 9.5 (in increments of 0.5 pH units). Amplification efficiency was evaluated at each pH to identify the optimal value.

### 2.17. Application to Pathogen Detection of the CL7-Bst Mutant

In order to confirm the feasibility of CL7-*Bst* mutant for practical pathogen detection, the real-time LAMP assay was carried out to evaluate detection efficiency and sensitivity for *E. coli* O157:H7. Total DNA of *E. coli* O157:H7 was extracted by two methods: a refined extraction (Section 2.3) and a crude Chelex 100 extraction. Refined extraction was performed as Section 2.3. Crude extraction using Chelex 100 was performed as follows: A 3 mL aliquot of an overnight *E. coli* O157:H7 culture was centrifuged at 12,000× *g* rpm for 2 min. The supernatant was discarded and the pellet retained. To the pellet, 200 μL of Chelex 100 (5% *w*/*v*) and 10 μL Proteinase K (20 mg/mL) were added, followed by incubation at 56 °C for 30 min. The sample was then heated at 99 °C in a metal bath for 10 min. After centrifugation at 13,000× *g* rpm for 10 min, the supernatant was collected as template DNA for subsequent detection.

Using the optimized LAMP conditions (from Section 2.11, Section 2.12, Section 2.13, Section 2.14, Section 2.15 and Section 2.16), we compared the detection performance of the CL7-*Bst* LAMP assay (primer information in Table S3) with a standard quantitative PCR (qPCR) assay (primer information in Table S4) for *E. coli* O157:H7. Each 20 μL qPCR reaction contained 10 μL of 2× ChamQ Universal SYBR qPCR Master Mix (Vazyme, Nanjing, China), 1 μL of forward primer (10 μM), 1 μL of reverse primer (10 μM), 2 μL of DNA template, and 6 μL of ddH_2_O. Amplification was performed with an initial denaturation at 95 °C for 5 min, followed by 40 cycles of 95 °C for 5 s, 56 °C for 30 s, and 72 °C for 40 s. Both crudely extracted DNA and refined DNA at concentrations of 1 × 10^2^, 1 × 10^3^, 1 × 10^4^, 1 × 10^5^, and 1 × 10^6^ CFU/mL were used as templates, with sterile ultrapure water as the negative control, to investigate the limit of detection (LOD) of the LAMP system.

## 3. Results

### 3.1. Screening and Expression of Target Bst DNA Polymerases

Using the DLKcat deep learning model, we predicted the *k_cat_* values of numerous *Geobacillus* polymerase sequences with strand-displacement activity. Sequences with the highest predicted *k_cat_* values were identified as A9X455 (6.2931), v5.9 (4.2426), A0A023CMU9 (2.2459), and A0A150MFP3 (0.2708) through the deep learning model (Figure S1). Sequence alignment demonstrated that A0A150MFP3 was 100% identical to *Bacillus stearothermophilus* (*Bst*) DNA polymerase (Figure S2).

**Figure 1:**
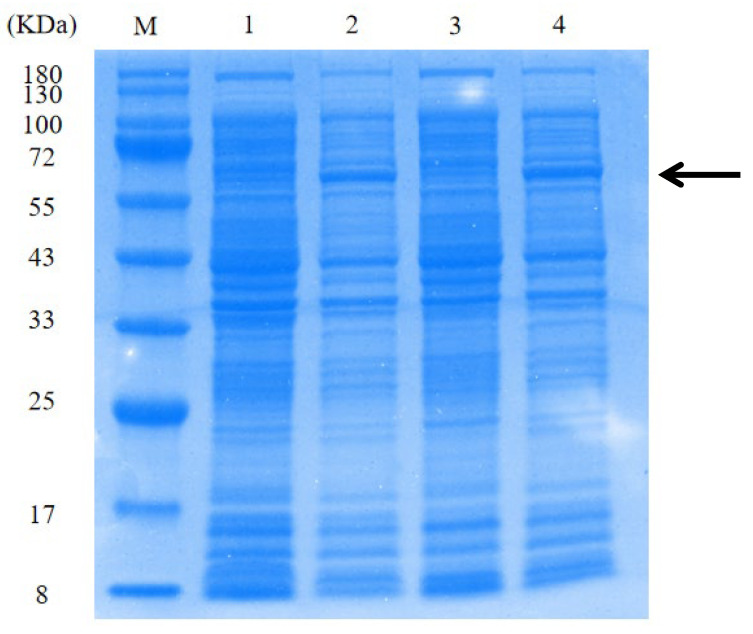
SDS-PAGE analysis of recombinant proteins. Lane M: protein marker; Lanes 1–4: A0A023CMU9, V5.9, A9X455 and *Bst*, respectively. The black arrows indicate the target.

Plasmids pET28a-*Bst*, pET28a-A9X455, pET28a-A0A023CMU9, and pET28a-v5.9 were constructed and induced for protein expression in *E. coli* BL21. Following cell lysis, supernatants were analyzed by SDS-PAGE (Figure 1). According to a protein statistics tool (http://www.detaibio.com/sms2/protein_stats.html, accessed on 22 August 2025), all four recombinant proteins were predicted to have a molecular weight of approximately 66 kDa. Strong bands at the expected 66 kDa position were observed for *Bst* and v5.9 (Figure 1), confirming successful expression of these proteins. In contrast, no band around 66 kDa was seen for A0A023CMU9 or A9X455, suggesting that these proteins were not expressed under the conditions used.

**Figure 2:**
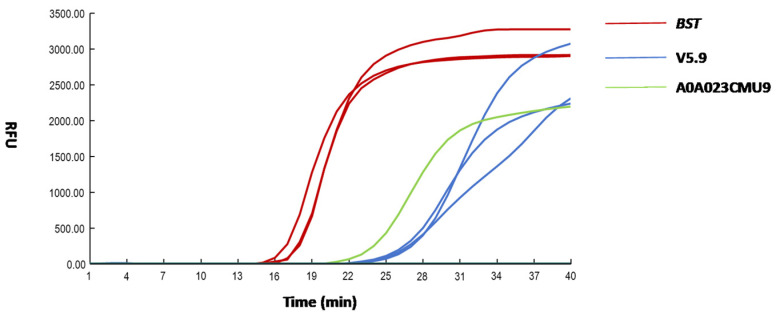
LAMP amplification of recombinant proteins. Red, blue, green bars indicate the performance of *Bst*, v5.9, A0A023CMU9, and A9X455, respectively.

The LAMP assay was used to evaluate the DNA polymerase activity of each recombinant protein (Figure 2). The threshold time (Tt) value of 18.20 was obtained for the *Bst*; the Tt value of 27.93 was obtained for the v5.9. The Tt value of 25.00 was obtained for the A0A023CMU9 with a single amplification curve, likely to be a false positive. No amplification curve was detected for the A9X455. These performance results correlated with the SDS-PAGE analysis: the absence of detectable expression for A9X455 and A0A023CMU9 corresponded to the lack of observed enzymatic activity.

Based on the study, the *Bst* was selected for subsequent molecular engineering studies.

### 3.2. Screening of Bst Mutant

To further explore the structure–function relationship of *Bst*, a 3D structural model was generated using the SWISS-MODEL online server (Figure S3). Using the *Bst* 3D model, two programs (COACH and HotSpot-Wizard) predicted a total of 20 potential functional sites (Tables S2 and S3). HotSpot-Wizard provided mutability scores and suggested beneficial mutations for its predicted hotspots. Meanwhile, COACH predicted that alanine substitutions at Arg288, Lys292, Tyr297, Arg325, Pro337, Ile338, Arg339, Gln507, and His539 would significantly alter ligand-binding affinity, indicating these residues are critical for enzyme function. We performed saturation mutagenesis on these key residues; variants predicted to improve binding affinity (ΔAffinity < −0.5) were noted (Table S4).

In total, 20 mutational hotspots were chosen for experimental testing, combining the suggestions from both COACH and HotSpot-Wizard. The mutational hotspots were: V32M, F81L, A86D, L105M, D119S, V120I, A122Q, S134H, K292R, N317S, A319C, N335R, N335K, N335Y, N335I, Q507K, Q507R, H539W, H539R, and H539K.

**Figure 3:**
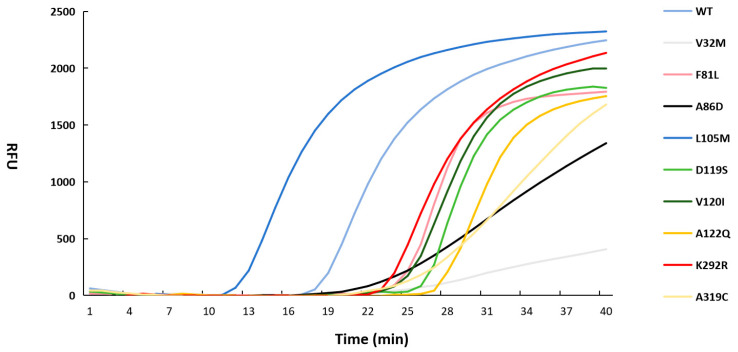
LAMP amplification of *Bst* mutant. The light blue curve indicates the amplification of *Bst* WT. The gray curve indicates the amplification of V32M. The light red curve indicates the amplification of F81L. The black curve indicates the amplification of A86D. The dark blue curve indicates the amplification of L105M. The light green curve indicates the amplification of D119S. The dark green curve indicates the amplification of V120I. The dark yellow curve indicates the amplification of A122Q. The dark red curve indicates the amplification of K292R. The light yellow curve indicates the amplification of A319C.

Performance of 20 *Bst* mutants was evaluated using the LAMP assay (Figure 3). The Tt value of 29.03 was obtained for the V32M, the Tt value of 24.38 was obtained for the F81L, the Tt value of 23.38 was obtained for the A86D, the Tt value of 12.47 was obtained for the L105M, the Tt value of 26.64 was obtained for the D119S, the Tt value of 25.64 was obtained for the V120I, the Tt value of 27.67 was obtained for the A122Q, the Tt value of 23.40 was obtained for the K292R, the Tt value of 25.21 was obtained for the A319C, the Tt value of 18.59 was obtained for the *Bst* WT. No amplification curve was detected for the remaining mutants.

L105M mutant exhibited a 32.92% increase in activity compared with WT enzyme. Therefore, L105M mutant was selected for subsequent experimental investigations.

### 3.3. Thermostability and Inhibitor Tolerance of the CL7-Bst Mutant

**Figure 4:**
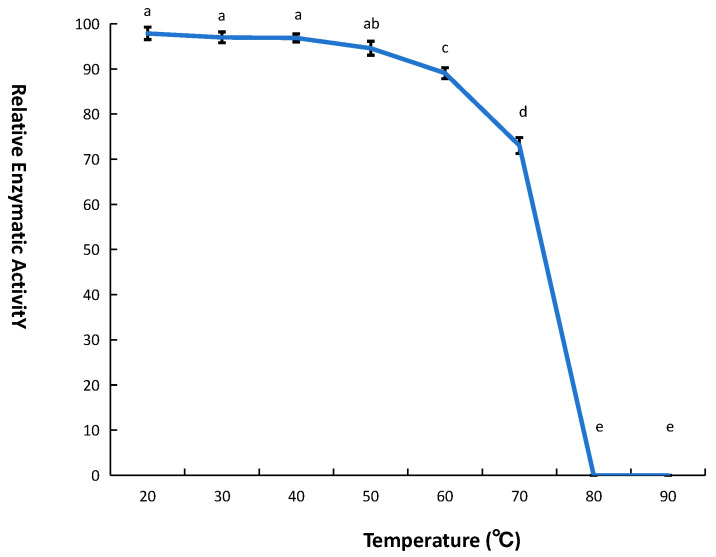
Thermostability of CL7-*Bst* mutant. The enzyme activity without incubation was set as 100%, and then the relative enzyme activity was calculated. Error bars represent the standard deviation (SD) of three replicates. Significant differences among groups (*p* < 0.05) are indicated by lowercase letters.

In this study, a CL7-*Bst* mutant was constructed by fusing the CL7 protein to the L105M mutant. The thermostability of the CL7-*Bst* mutant was evaluated by measuring enzymatic activity. The thermostability was assessed at various temperatures. Results indicated that the CL7-*Bst* mutant exhibited superior thermostability compared with wild-type *Bst* within the temperature range of 0–70 °C. It was found that the enzymatic activity of the CL7-*Bst* mutant remained stable and active for 2 h at 0–50 °C, the relative enzyme activity decreased to 90% after 2 h of incubation at 60 °C, the relative enzymatic activity decreased to 70% after 2 h of incubation at 70 °C, the polymerase was completely inactivated after 2 h of incubation at 80 °C (Figure 4).

**Figure 5:**
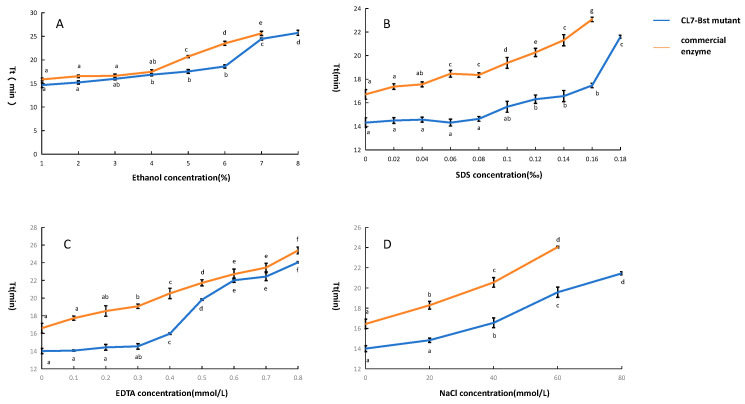
Inhibitor tolerance of CL7-*Bst* mutant and commercial enzyme. (**A**) Ethanol tolerance of CL7-*Bst* mutant and commercial enzyme. (**B**) SDS tolerance of CL7-*Bst* mutant and commercial enzyme. (**C**) NaCl tolerance of CL7-*Bst* mutant and commercial enzyme. (**D**) EDTA tolerance of CL7-*Bst* mutant and commercial enzyme. The blue curve indicates the threshold time (Tt) of CL7-*Bst* mutant. The orange curve indicates the threshold time of commercial enzyme. Error bars represent the standard deviation (SD) of three replicates. Significant differences among groups (*p* < 0.05) are indicated by lowercase letters.

The inhibitor tolerance of CL7-*Bst* mutant was evaluated by LAMP. Results of inhibitor tolerance indicated that the CL7-*Bst* mutant exhibited better tolerance up to 7% (*v*/*v*) ethanol, while the commercial enzyme showed no amplification curve at the same ethanol concentration (Figure 5A). The CL7-*Bst* mutant exhibited better tolerance up to 0.18‰ (*w*/*v*) SDS, while the commercial enzyme showed no amplification curve at the same SDS concentration (Figure 5B). The CL7-*Bst* mutant exhibited better tolerance up to 80 mmol/L NaCl, while the commercial enzyme showed no amplification curve at the same NaCl concentration (Figure 5C). Both CL7-*Bst* mutant and the commercial enzyme maintained amplification at up to 0.8 mmol/L EDTA; however, CL7-*Bst* was more stable than the commercial enzyme within the 0–0.4 mmol/L concentration (Figure 5D).

### 3.4. Optimization of the LAMP Reaction System with the CL7-Bst Mutant

**Figure 6:**
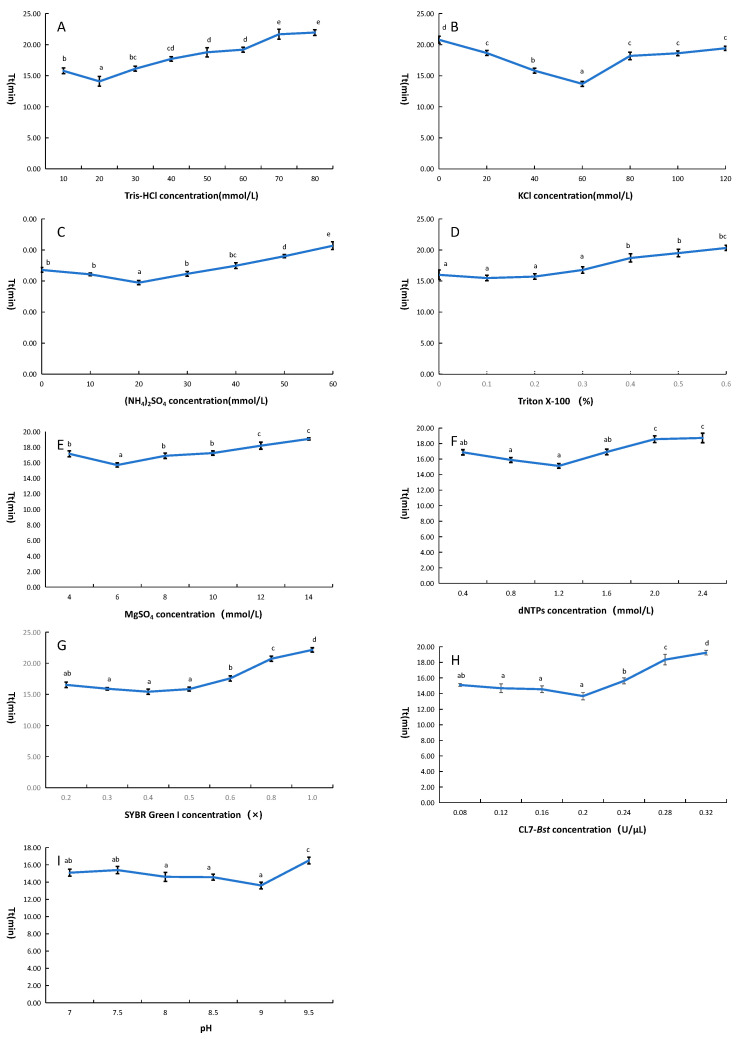
Optimization of CL7-*Bst* Mutant-Based LAMP Reaction System. (**A**) Optimization of Tris-HCl concentration. (**B**) Optimization of KCl concentration. (**C**) Optimization of (NH4)_2_SO_4_ concentration. (**D**) Optimization of Triton X-100 concentration. (**E**) Optimization of Triton MgSO_4_ concentration. (**F**) Optimization of dNTPs concentration. (**G**) Optimization of SYBR Green I concentration. (**H**) Optimization of CL7-*Bst* concentration. (**I**) Optimization of pH. Tt indicates threshold time. Error bars represent the standard deviation (SD) of three replicates. Significant differences (*p* < 0.05) are indicated by lowercase letters.

The LAMP reaction system using CL7-*Bst* mutant was optimized by adjusting the concentrations of Tris-HCl, KCl, (NH_4_)_2_SO_4_, Triton X-100, Mg^2+^, dNTPs, SYBR Green I, CL7-*Bst* mutant and adjusting pH. Within the tested range of Tris-HCl concentrations (10–80 mmol/L), the lowest threshold time was observed at 20 mmol/L (Figure 6A). Within the tested range of KCl concentrations (0–140 mmol/L), the lowest threshold time was observed at 60 mmol/L (Figure 6B). Within the tested range of (NH_4_)_2_SO_4_ concentrations (0–60 mmol/L), the lowest threshold time was observed at 20 mmol/L (Figure 6C). Within the tested range of Triton X-100 concentrations (0.1–0.6%), the lowest threshold time was observed at 0.1% (Figure 6D). Within the tested range of Mg^2+^ concentration (4–14 mmol/L), the lowest threshold time was observed at 6 mmol/L (Figure 6E). Within the tested range of dNTP concentrations (0.4–2.4 mmol/L), the lowest threshold time was observed at 1.2 mmol/L (Figure 6F). Within the tested range of SYBR Green I concentrations (0.2×–1×), the lowest threshold time was observed at 0.4× (Figure 6G). Within the tested range of CL7-*Bst* mutant concentrations (0.08–0.32 U/μL), the lowest threshold time was observed at 0.20 U/μL (Figure 6H). Within the tested range of pH (7.0–9.5), the lowest threshold time was observed at 9.0 (Figure 6I).

### 3.5. Optimization of the Reaction Temperature for the CL7-Bst Mutant

**Figure 7:**
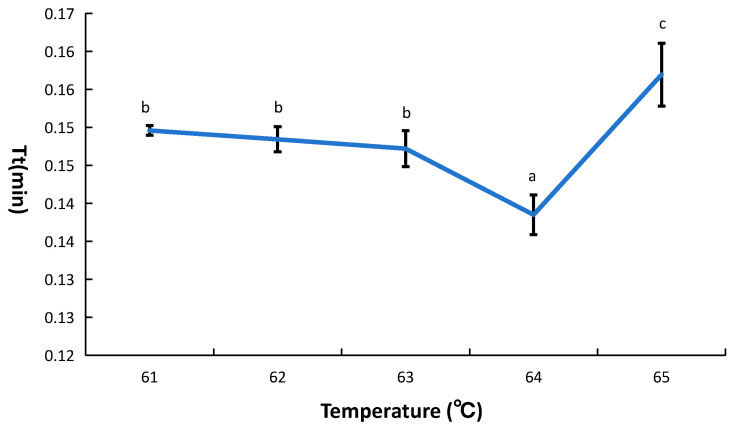
Optimization of temperature. Tt indicates threshold time. Error bars represent the standard deviation (SD) of three replicates. Significant differences among groups (*p* < 0.05) are indicated by lowercase letters.

To optimize the LAMP reaction system temperature, five temperature gradients (61 °C, 62 °C, 63 °C, 64 °C, and 65 °C) were tested. The lowest threshold time was observed at 64 °C (Figure 7). The temperature of the LAMP reaction system is known to affect primer binding and the enzymatic activity of *Bst* DNA polymerase. At suboptimal temperatures, primer dimers may readily form, and full enzymatic activity may not be achieved. Conversely, excessively high temperatures can compromise primer annealing and lead to reduced enzymatic activity. The results indicated that optimal enzymatic activity was achieved at 64 °C.

### 3.6. Amplification Capacity of the CL7-Bst Mutant in LAMP Reaction System for E. coli O157:H7

**Figure 8:**
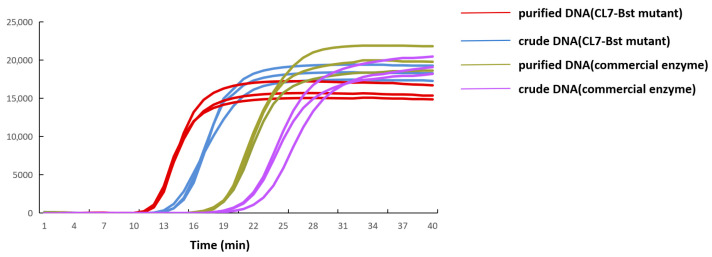
Comparison of LAMP reaction using CL7-*Bst* mutant and commercial enzyme with crude and purified *E. coli* O157:H7 DNA. Red and blue curves: CL7-*Bst* with purified and crude DNA, respectively. Green and purple curves: commercial enzyme with purified and crude DNA, respectively.

**Figure 9:**
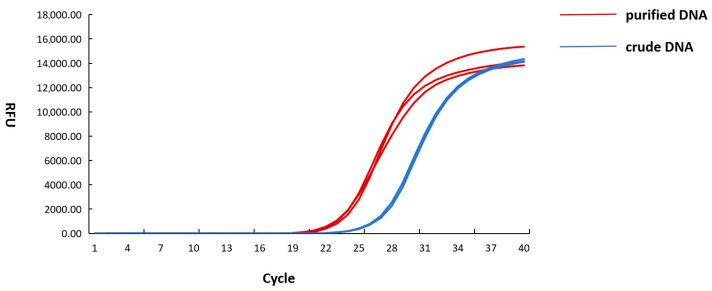
Real-time qPCR amplification curves for *E. coli* O157:H7 DNA. Red: purified DNA; Blue: crude DNA.

LAMP reactions were performed to evaluate the amplification capacity of CL7-*Bst* mutant for the detection of *E. coli O157:H7*. Both DNA extracted by refined extraction and crude extraction were used in this study. Results obtained using commercial enzyme and quantitative PCR assay were employed to systematically evaluate the detection efficacy of the CL7-*Bst* mutant. In the LAMP reaction system using CL7-*Bst* mutant, a Tt value of 15.13 was observed in crude DNA and 12.78 in purified DNA. In the LAMP reaction system using commercial enzyme, a Tt value of 22.03 was observed in crude DNA and 18.56 in purified DNA (Figure 8). In the quantitative PCR assay, a Ct value of 27.56 was observed in crude DNA and 23.76 in purified DNA (Figure 9). It should be noted that quantitative PCR assay required over 60 min, while LAMP reaction system was completed within 40 min without thermal denaturation or cycling steps.

**Figure 10:**
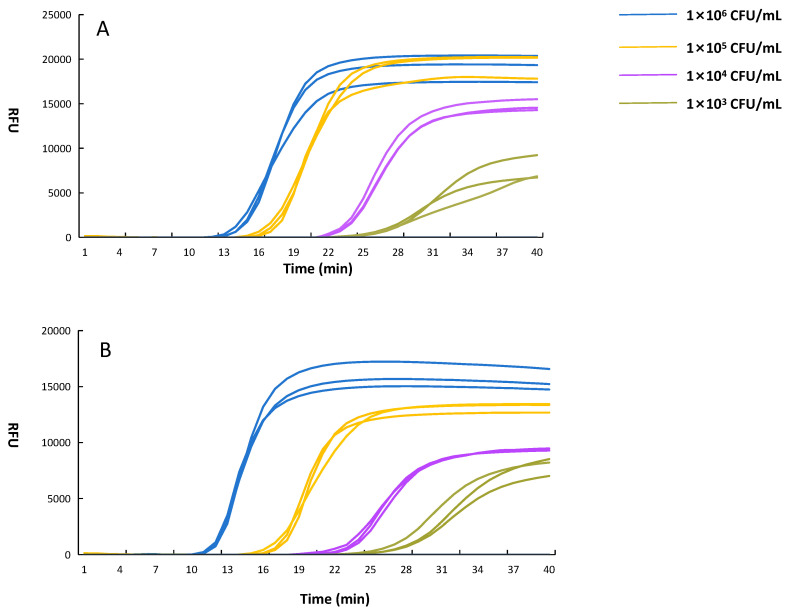
Limit of detection of the CL7-*Bst* LAMP system. (**A**) Crude DNA template; (**B**) Purified DNA template. The curves correspond to 1 × 10^6^ (blue), 1 × 10^5^ (yellow), 1 × 10^4^ (purple), 1 × 10^3^ (green).

In addition, limit of detection (LOD) of LAMP reaction system using CL7-*Bst* mutant for *E. coli* O157:H7 was determined in this study. Both crude DNA and purified DNA were diluted from 1 × 10^2^ to 1 × 10^6^ CFU/mL. No amplification was observed at 1 × 10^2^ CFU/mL for either crude DNA or purified DNA. Detectable amplification was observed down to 1 × 10^3^ CFU/mL for both crude DNA and purified DNA, suggesting that this LAMP reaction system maintained high sensitivity even when crude extraction was used (Figure 10A,B). These results indicate that the optimized CL7-*Bst* LAMP system can significantly improve the speed and sensitivity of on-site foodborne pathogen detection.

## 4. Discussion

The engineering of *Bst* DNA polymerase with improved catalytic efficiency and robustness is of considerable importance for expanding the practical utility of LAMP-based detection systems. In the present study, we combined deep learning with semi-rational design to engineer *Bst* DNA polymerase. This workflow enabled the rapid identification of a beneficial mutation and the construction of a CL7-*Bst* mutant with superior thermostability and inhibitor tolerance. The results highlight the value of combining computational prediction with experimental verification in enzyme engineering, particularly for accelerating the development of polymerases suitable for rapid and on-site molecular diagnostics.

*k_cat_* serves as a crucial parameter for understanding an organism’s metabolism, proteome allocation, growth, and physiology [20,21]. Using the DLKcat deep learning model, this study predicted the *k_cat_* values of numerous *Geobacillus* polymerase sequences. It was previously reported that the *Bst* fragment originates from *Geobacillus stearothermophilus* GIM1.543 [22]. The results of this study confirm that A0A150MFP3 is in fact the *Bst* large fragment gene from *G. stearothermophilus* GIM1.543. Nonetheless, although the DLKcat model has been validated in *E*. *coli*, yeast, and fungi [16], certain deviations may occur when applying this model to other species. Therefore, predictions for *Geobacillus* enzymes may have larger errors. In this study, the deep learning model was mainly used to narrow the range of candidate enzymes, whereas the final selection still relied on subsequent experimental screening and validation. Although some candidates showed higher predicted *k_cat_* values, they were not successfully obtained under the current expression conditions. We thus proceeded to experimentally validate these candidates rather than relying solely on in silico data.

Isothermal amplification reactions require DNA polymerases to possess strong stability to ensure normal reaction progression. Enhanced thermostability of DNA polymerase correlates with greater reaction sustainability; therefore, investigating the thermostability of DNA polymerases is of significant importance. It has been reported that the wild-type *Bst* retains only 80% activity after 2 h at 60 °C, declining to 35% after 2 h at 70 °C [23]. The CL7-*Bst* mutant maintained activity until incubation at 80–90 °C for 2 h, demonstrating significantly enhanced thermostability. However, because fused and unfused forms with and without the L105M substitution were not compared side by side, the respective contributions of the mutation and the fusion partner to the thermal improvement could not be separately evaluated in this study.

Residual substances from food additives, sample preparation, and nucleic acid extraction processes are often found to inhibit nucleic acid amplification reactions. Therefore, the tolerance of DNA polymerases to common inhibitors should be rigorously evaluated. Enhanced inhibitor tolerance is considered to be directly associated with the reliability of on-site detection. During sample preparation and nucleic acid extraction, ethanol residues are commonly observed and have been reported to interfere with amplification reactions [24]. SDS, as a typical component of lysis buffer, has been shown to significantly reduce amplification efficiency when present as a residual contaminant [25]. NaCl, frequently used as a food additive in processed foods, is known to suppress amplification reactions at elevated concentrations [26]. EDTA is commonly employed during nucleic acid extraction to stabilize DNA and prevent degradation [27]. In this study, these four common inhibitors—ethanol, SDS, NaCl, and EDTA—were selected to evaluate the inhibitor tolerance of the CL7-*Bst* mutant during amplification. Our results confirm that the CL7-*Bst* mutant has markedly improved tolerance to these inhibitors, which is beneficial for field applications with minimally processed samples. As with thermostability, the improved inhibitor tolerance observed here reflects the overall performance of the engineered construct, and the individual effects of L105M and CL7 were not distinguished.

Besides engineering the enzyme itself, systematic optimization of the LAMP reaction system was also necessary to fully exploit the performance of the CL7-*Bst* mutant. Optimization of the LAMP reaction system is primarily achieved through modified primer design [28]. However, existing optimizations of reaction systems typically rely on empirical adjustments, often without consideration of the potential need for enzyme-specific reaction conditions. In this study, the LAMP reaction system with the CL7-*Bst* mutant was systematically optimized by modulating the pH, the concentrations of Tris-HCl, KCl, (NH_4_)_2_SO_4_, Triton X-100, Mg^2+^, dNTPs, SYBR Green I, and the CL7-*Bst* mutant. Real-time LAMP was used to compare the amplification performance of the engineered polymerase. Compared with conventional endpoint LAMP, real-time LAMP can monitor the amplification process continuously and provide the threshold time (Tt). This makes it more convenient for comparing reaction efficiency under different conditions.

The practical value of the optimized CL7-*Bst* system was demonstrated using *E. coli* O157:H7 as a model foodborne pathogen. Under the optimized conditions, the CL7-*Bst*-based LAMP assay showed lower Tt values than the commercial enzyme for both crude and purified DNA templates, while maintaining a detection limit of 1 × 10^3^ CFU/mL. In addition, the assay could be completed within 40 min, whereas qPCR required more than 60 min. These results indicate that the engineered polymerase and optimized reaction system can improve both speed and sensitivity for pathogen detection, even when crude DNA templates are used. Such performance is particularly valuable for food microbiology, rapid screening, and field-based surveillance, where short turnaround time and simplified operation are highly desirable. At the same time, the commercial comparison in this study was limited, and inclusion of other widely used *Bst* derivatives would allow a broader evaluation of the practical performance of the engineered enzyme.

Non-specific amplification is a common problem in LAMP assays. In this study, the main focus was on the activity, thermostability, and inhibitor tolerance of the CL7-*Bst* mutant. The effect of the CL7-*Bst* mutant on non-specific amplification was not further evaluated. This issue still needs to be investigated in future studies.

Overall, this study demonstrates that combining deep learning and semi-rational design for *Bst* DNA polymerase engineering, together with reaction system optimization, is an effective strategy for improving the performance of *Bst* DNA polymerase for microbial detection. The resulting CL7-*Bst* mutant showed enhanced enzymatic activity, thermostability, and inhibitor tolerance, while the optimized LAMP system enabled rapid and sensitive detection of *E. coli* O157:H7 from both purified and crude DNA templates. Further work will be helpful to evaluate prediction accuracy within this enzyme family more systematically, to distinguish the effects of the L105M substitution and CL7 fusion more clearly, and to compare the engineered enzyme with a wider range of commercial *Bst* derivatives. These findings provide not only a promising engineered polymerase for LAMP-based detection of foodborne pathogens, but also a useful framework for the development of robust and field-deployable molecular tools in microbiological diagnostics.

## 5. Conclusions

In this study, a strategy combining a deep learning model with semi-rational design was used for candidate screening and enzyme engineering. A0A150MFP3 was selected for subsequent study, and the L105M mutation increased enzyme activity by 32.92% compared with the wild type. Based on this mutant, the CL7 protein was fused for isothermal amplification, generating a CL7-*Bst* mutant with excellent thermal stability and tolerance to 7% (*v*/*v*) ethanol, 0.18‰ (*w*/*v*) SDS, 80 mmol/L NaCl, and 0.8 mmol/L EDTA. The LAMP reaction system with the CL7-*Bst* mutant was subsequently optimized, determining the reaction pH to be 9.0, the optimal enzyme concentration to be 0.20 U/μL, and the reaction temperature to be 64 °C. When applied to the detection of *E. coli* O157:H7, the Tt values obtained from DNA by crude extraction and DNA by purified extraction were 15.13 and 12.78, with a detection limit of 1 × 10^3^ CFU/mL for both. In conclusion, the CL7-*Bst* mutant showed good performance in rapid nucleic acid detection and has potential for practical application in rapid detection of foodborne pathogens.

## Data Availability

The original contributions presented in this study are included in the article. Further inquiries can be directed to the corresponding authors.

## Supplementary Materials

The following supporting information can be downloaded at: https://www.mdpi.com/article/10.3390/microorganisms14050954/s1, Figure S1. Phylogenetic tree of protein sequence; Figure S2. Result of protein sequence alignment; Figure S3. Wild-type *Bst* 3D structural model; Table S1. Primers in this study; Table S2. Results of COACH; Table S3. Results of HotSpot-Wizard; Table S4. Result of Dicovery Studio.
