# Supplementary figures and images for: Deep Learning-Guided Engineering of Bst DNA Polymerase Improves LAMP-Based Detection of Foodborne Pathogens
Source: Microorganisms. 2026 Apr 23;14(5):954. doi: 10.3390/microorganisms14050954 (PMC13209251; doi:10.3390/microorganisms14050954)

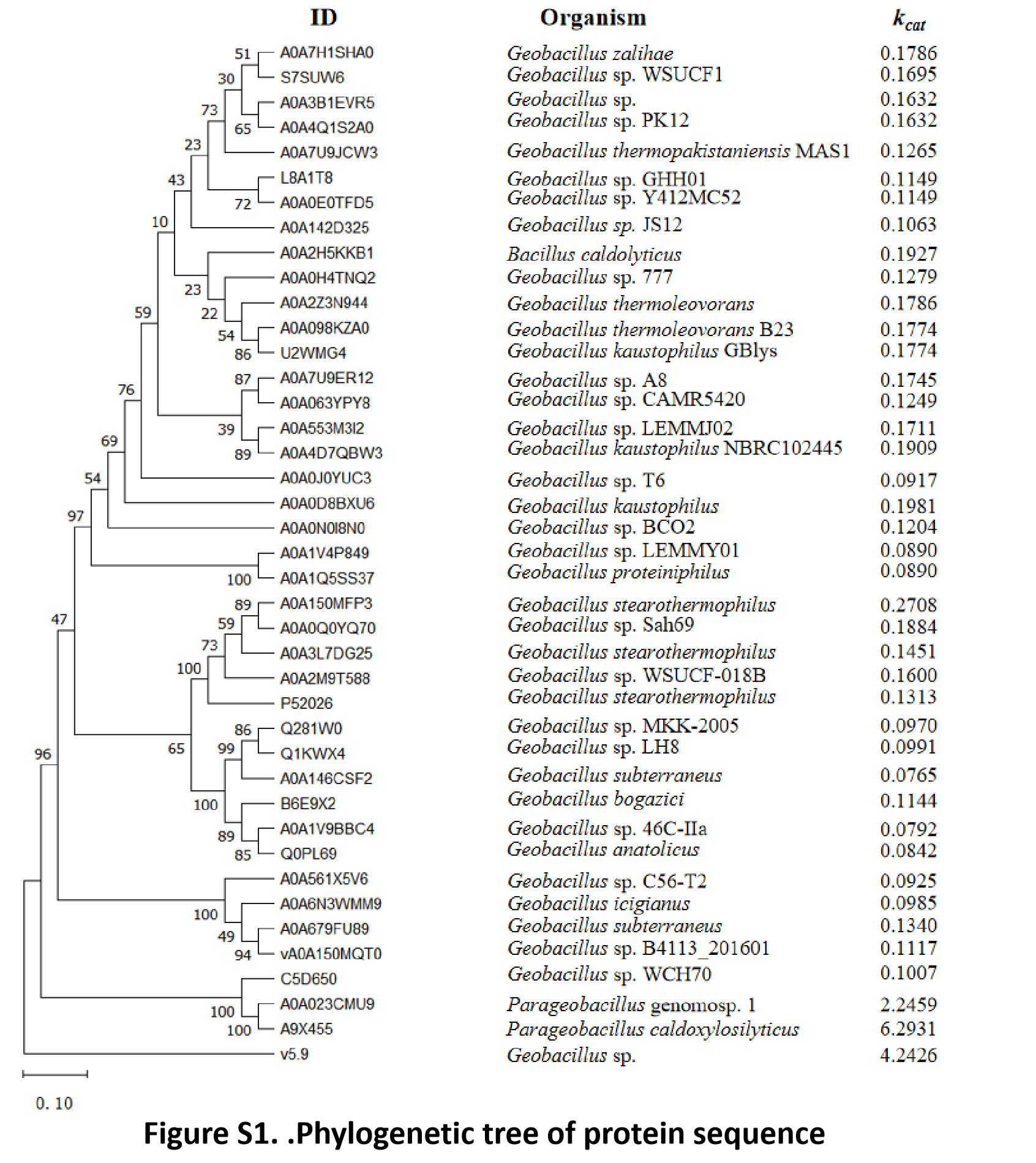

Supplement: Supplementary file 1 [file microorganisms-14-00954-s001.zip › sup fig/Fig.S1.png]

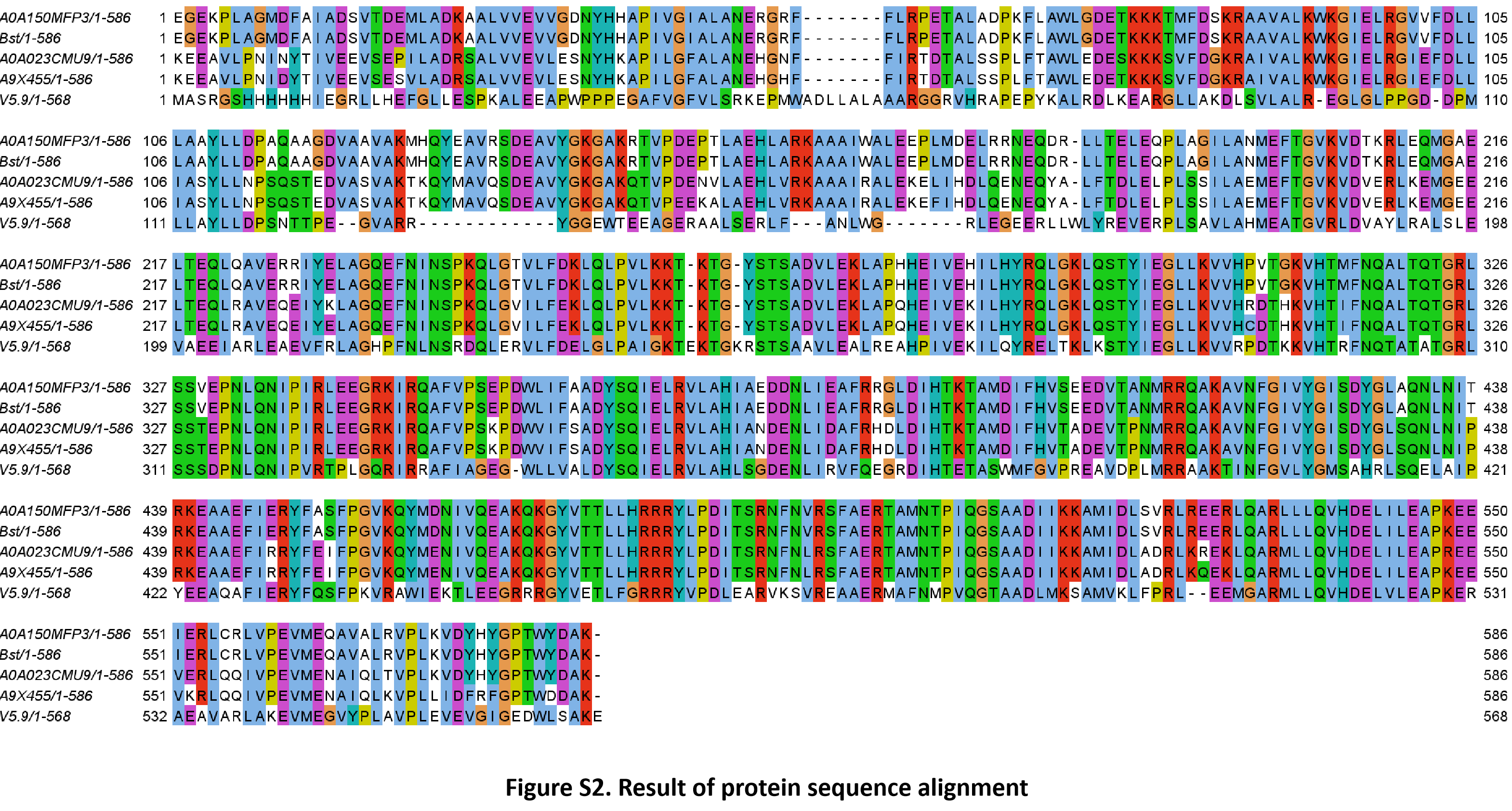

Supplement: Supplementary file 1 [file microorganisms-14-00954-s001.zip › sup fig/Fig.S2.png]

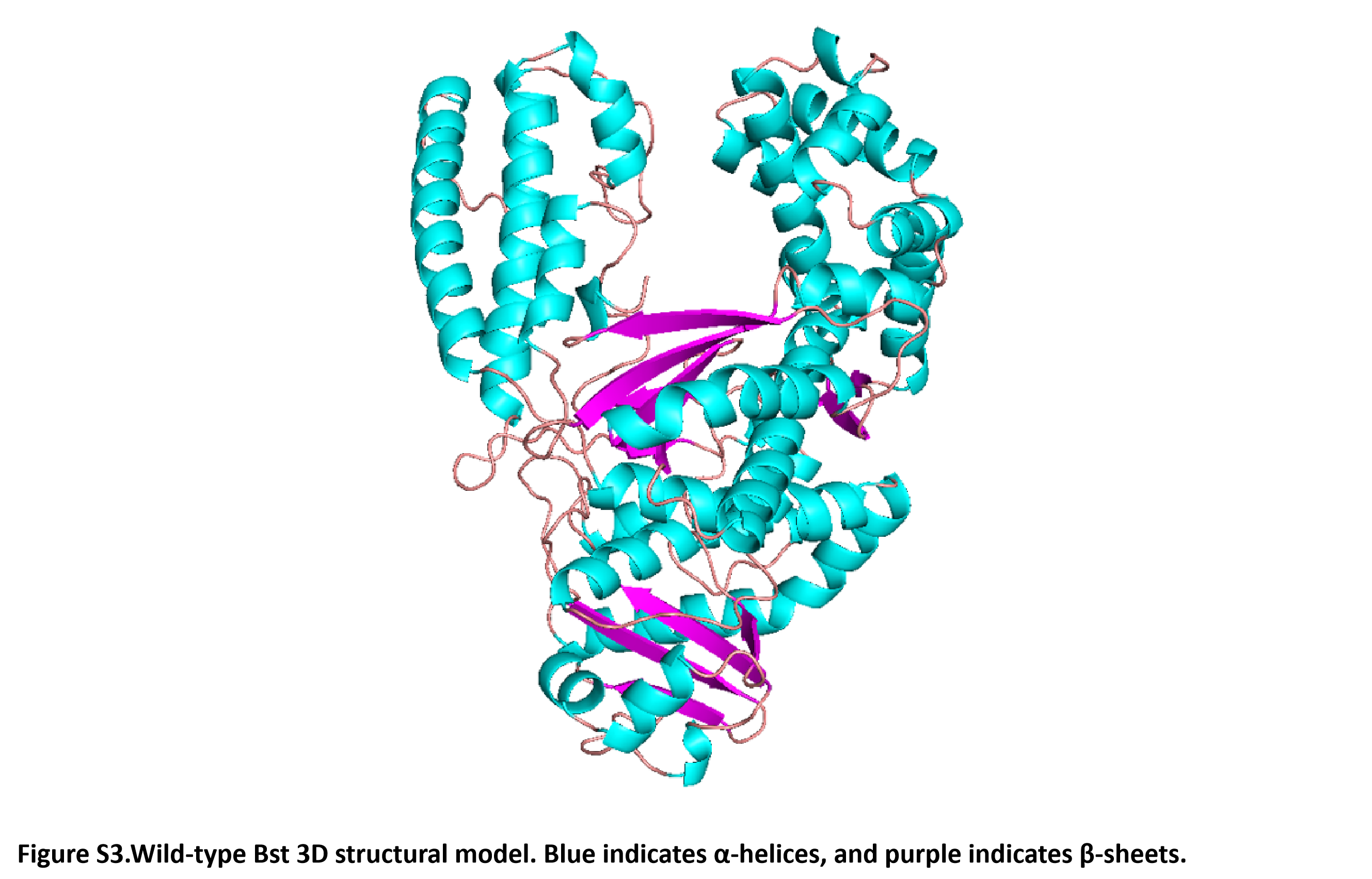

Supplement: Supplementary file 1 [file microorganisms-14-00954-s001.zip › sup fig/Fig.S3.png]
